# Diet Quality Is Not Associated with Malnutrition, Low Muscle Mass and Sarcopenia During Lung Cancer Treatment: A Cross-Sectional Study †

**DOI:** 10.3390/nu18050764

**Published:** 2026-02-26

**Authors:** Annie R. Curtis, Nicole Kiss, Robin M. Daly, Gavin Abbott, Anna Ugalde, Katherine M. Livingstone

**Affiliations:** 1 Institute for Physical Activity and Nutrition (IPAN), School of Exercise and Nutrition Sciences, Deakin University, Geelong, VIC 3220, Australia; nicole.kiss@deakin.edu.au (N.K.); robin.daly@deakin.edu.au (R.M.D.); gavin.abbott@deakin.edu.au (G.A.); k.livingstone@deakin.edu.au (K.M.L.); 2 Allied Health Research, Peter MacCallum Cancer Centre, Melbourne, VIC 3000, Australia; 3 School of Nursing and Midwifery, Centre for Quality and Patient Safety in the Institute for Health Transformation, Deakin University, Geelong, VIC 3220, Australia; a.ugalde@deakin.edu.au

**Keywords:** lung cancer, dietary patterns, malnutrition, low muscle mass, sarcopenia

## Abstract

**Background/Objectives**: Studies evaluating the impact of diet quality on nutrition- and muscle-related outcomes in cancer are limited. This study aimed to understand the diet quality of people with lung cancer and its cross-sectional associations with malnutrition, low muscle mass and (probable)-sarcopenia. **Methods**: Three-day food records were collected from 47 adults (mean ± SD age 70.6 ± 8.6 years; 58% male) with lung cancer prior to, or within one week, of curative-intent (chemo)radiotherapy. Dietary Guidelines Index (DGI-2013) and Mediterranean Diet Score (MDS) estimated diet quality, reflecting established healthy eating patterns. Malnutrition was determined using Patient Generated Subjective Global Assessment (PG-SGA). Low muscle mass was estimated using diagnostic third lumbar vertebra computed tomography (CT) images. (Probable)-sarcopenia was determined using the revised European Working Group for Sarcopenia in Older People definition, including low muscle (grip) strength, muscle mass and impaired function. Multivariate adjusted logistic regression analyses estimated odds ratios (OR) and 95% confidence intervals (CI) for associations between diet quality and outcomes. **Results**: Prevalence of malnutrition, low muscle mass and (probable)-sarcopenia were 36.2%, 50.0% and 13.6%, respectively. Mean ± SD DGI-2013 score was 53.0 ± 13.0. Adherence to the DGI-2013 was not significantly associated with malnutrition (OR, 0.67 [95%CI 0.35, 1.28]), low muscle mass (0.90 [95%CI 0.47, 1.70]) or (probable)-sarcopenia (0.73 [95%CI 0.29, 1.80]). Mean ± SD MDS was 3.6 ± 1.5. Adherence to the MDS was not significantly associated with malnutrition (0.75 [95%CI 0.37, 1.49]), low muscle mass (0.98 [95%CI 0.51, 1.88]) or (probable)-sarcopenia (1.82 [95%CI 0.72, 4.85]). **Conclusions**: Diet quality was not associated with malnutrition, low muscle mass or (probable)-sarcopenia. Given that overall diet quality was poor, it remains unclear whether high diet quality may be associated with nutritional status or muscle-related outcomes. Further research is needed to determine whether diet quality should be considered in nutritional interventions during lung cancer treatment.

## 1. Introduction

Lung cancer is the second most commonly diagnosed cancer, accounting for 11% of diagnoses worldwide [1]. Despite recent advancements in treatment modalities, lung cancer remains the leading cause of cancer-related deaths, responsible for 1.8 million deaths in 2020 [1]. Half of all people with lung cancer experience malnutrition or low muscle mass [2,3], which can contribute to poorer quality of life and an increased risk of morbidity and mortality [4,5]. People with lung cancer are also at risk of sarcopenia, characterized by low muscle strength, low muscle mass and impaired physical function [6]. While it has not been extensively examined among people with lung cancer, sarcopenia has been shown to result in an up to three-fold increased risk of mortality in adults with other cancer types [4]. Therefore, effectively preventing and/or managing malnutrition, low muscle mass and sarcopenia is essential for improving lung cancer outcomes.

Adequate nutrition and targeted nutritional therapies play a fundamental role in all stages of lung cancer treatment. Evidence-based nutrition guidelines advocate for the optimisation of energy and protein intake to address the high risk of malnutrition, low muscle mass and sarcopenia among people with cancer [7,8]. However, there are currently no lung-cancer-specific nutritional guidelines, and the potential role of overall diet quality, beyond individual nutrient targets, in managing outcomes during cancer treatment is not well understood.

Dietary patterns are emerging as a potential approach to managing malnutrition, low muscle mass and sarcopenia, considering the overall composition and frequency of food and beverage intakes, rather than isolated nutrients [9]. Dietary patterns aligned with national dietary guidelines or Mediterranean dietary principles are characterised by high intakes of fruits, vegetables, whole grains, and unsaturated fats, and lower intakes of discretionary foods. Previous studies have demonstrated the propensity for people to modify their dietary patterns after a cancer diagnosis [10,11]. For example, survivors may aim to improve diet quality and align their intake to such recommendations, reducing intake of red meats, sugar and fat-containing foods, whilst increasing intake of fruits and vegetables [11]. High-quality dietary patterns have demonstrated benefits for quality of life and fatigue in people with cancer [12]. However, few studies have purposefully examined the impact of high-quality dietary patterns on nutritional status and muscle-related outcomes in lung cancer. Therefore, the aim of this study was to examine the diet quality of people with lung cancer using a priori dietary pattern methods and assess their associations with malnutrition, low muscle mass and sarcopenia.

## 2. Materials and Methods

### 2.1. Study Design and Participants

This study used data from the PREDICT study, Predicting Muscle Loss During Lung Cancer Treatment, a prospective observational cohort that aimed to identify predictors of loss of muscle mass and radiodensity (increased muscle fat infiltration) in people with lung cancer following curative-intent (chemo)radiotherapy. The PREDICT study protocol has been described elsewhere [13]. In brief, data were collected across three timepoints: (i) prior to or within one week of (chemo)radiotherapy (baseline, T1); (ii) during the final week of (chemo)radiotherapy (T2), and (iii) two months after completion of (chemo)radiotherapy (T3). Data collection occurred between September 2019 and April 2023. For the present analysis, only data collected at T1 were examined.

Adults were eligible for inclusion if they were scheduled to receive curative-intent, standard-dose, and fraction (chemo)radiotherapy for non-small cell lung cancer (NSCLC) or small cell lung cancer (SCLC) at one of three tertiary health services in Victoria, Australia. Exclusion criteria included: (i) evidence of cognitive impairment or a psychiatric condition; (ii) conditions known to affect body composition (e.g., human immunodeficiency virus or muscular dystrophy); or (iii) recent diagnosis of thyroid disease (less than three months). Non-English-speaking patients were eligible if they could give informed consent with family interpretation. Participants without dietary data at T1 were excluded from the analysis. A flow diagram of eligible participants is presented in Figure S2.

Written informed consent was provided by all participants prior to participation. The PREDICT study received ethical approval from Peter MacCallum Cancer Centre Human Research Ethics Committee (HREC/53147/PMCC-2019) on 19 June 2019. This article is a revised and expanded version of a conference abstract entitled “Diet quality is not associated with malnutrition, low muscle mass and sarcopenia during lung cancer treatment” which was presented at the Clinical Oncology Society of Australia Annual Scientific Meeting, 13–15 November 2024 [14].

### 2.2. Data Collection and Study Measures

#### 2.2.1. Dietary Intake

Dietary intake was collected using hard-copy three-day food records by participants in their homes and, following their return, transposed into the Automated Self-Administered 24 h (ASA24) online dietary assessment tool by researchers [15]. Data entry was completed by two researchers who were trained in the use of ASA24, following a data entry protocol. This multi-step method minimised participant burden, negating the need for digital device access. A list of ‘assumptions’ (commonly consumed food types) was used to ensure consistent reporting between researchers when insufficient information about dietary intake was provided by the participants and when the participant could not be contacted for clarification. Dietary data from two weekdays and one weekend day were collected in the week immediately after the assessment visit, with verbal instructions on how to complete the food diary and visual aids provided for accurate portion size estimation. Food amount (grams/day), energy and nutrient intakes were computed from foods and beverages reported by participants. The ASA24 Australia 2016 is adapted to reflect the Australia food supply and utilises the Australian Food, Supplement, and Nutrient Database (AUSNUT) 2011 to 2013 food and measure codes for nutrient estimations. Standard portion sizes were derived from the 2011 to 2013 Australian Health Survey [15].

#### 2.2.2. Dietary Patterns

Dietary data were used to estimate adherence to the Dietary Guideline Index 2013 (DGI-2013) [16] and Mediterranean Diet Score (MDS) [17,18]. The DGI-2013 reflects alignment with the current Australian Dietary Guidelines, and the MDS represents adherence to the Mediterranean diet, a well-established high-quality dietary pattern.

### 2.3. The Dietary Guideline Index-2013

The DGI-2013 is a food-based score designed to estimate adherence to the current Australian Dietary Guidelines [19]. The DGI-2013 includes 13 components: seven recommended (food variety, fruits, vegetables, cereals, meat and alternatives, dairy and alternatives, and beverage intake) and six discouraged (discretionary foods, foods high in saturated fat, low in unsaturated fat, added sugars, added salt, and alcohol intake). For the present analysis, trimmed meat (a sub-component of saturated fat) and salt intake were excluded due to limited data availability (Table S1).

Total scores ranged from 0 to 115, with a higher score indicating closer adherence to Australian Dietary Guidelines and better overall diet quality. Components were scored out of 10 and sub-components (cereals, meat and alternatives, beverages, and saturated fats) were scored out of five (Table S1). Cut-offs for maximum scores were based on age- and sex-specific recommendations from the Australian Dietary Guidelines [20] and were achieved when average daily consumption met or exceeded the recommendation. A score of zero indicated non-adherence. Intake of recommended components between the minimum and maximum criteria resulted in proportionate scores. Proportional scores were not used for discouraged dietary components [21,22].

Ratios were used to score sub-components of cereal (wholegrain/wholemeal bread:total bread), meat and alternatives (lean meat:total meat), beverage intake (water:total fluid intake), and saturated fat intake (low-fat milk:total milk intake). Food variety, measuring variety within five encouraged food groups (vegetables, fruits, meat and alternatives, dairy and alternatives, and cereals), was scored using established cut-off points (>15 g/day, beverages; and >20 g/day, foods) [23,24]. Scores for each food group (1 point each) were summed and divided by the total number of foods within each group. A total food variety score (out of 10) was generated by summing scores across the five food groups and multiplying by two [23,24].

### 2.4. Mediterranean Diet Score

The MDS 9-item food- and nutrient-based index was designed to estimate adherence to the Mediterranean diet. Developed and validated by Trichopoulou et al. [17,18], the MDS is the first and most frequently used Mediterranean diet index. Food and nutrient intakes were scored against nine components: vegetables, legumes, fruits and nuts, cereals, fish and seafood, monounsaturated-fat-to-saturated-fat ratio, dairy products, meat and meat products, and alcohol (Table S2).

Scores for each component were summed so that the total MDS ranged from 0 to 9, with higher scores reflecting closer adherence to the Mediterranean diet. Components were scored using sex-specific median intakes as cut-offs [18]. A score of 1 was allocated when intakes of vegetables, legumes, fruits and nuts, cereals, fish and seafood, and the ratio of monounsaturated:saturated fats were equal to or above the median, or when intake of dairy products and meat and meat products was below the median. For alcohol, a score of 1 was allocated for low-to-moderate intake (no more than 2 times/day) or zero when intake was greater than 2 times/day or no intake [25]. A serving of alcohol or discretionary foods was defined as 600 kJ in line with a discretionary serving from the Australian Dietary Guidelines [19]. Due to a large proportion of non-consumers of legumes and fish and seafood (i.e., participants who reported not consuming these food groups), the median intake of ‘consumers’ was utilised for these components.

### 2.5. Malnutrition

Malnutrition was assessed using the Patient Generated Subjective Global Assessment (PG-SGA) [26] which is validated for use in patients undergoing radiotherapy [27,28]. Patients were categorised as A—well nourished, B—mild to moderately malnourished, and C—severely malnourished.

### 2.6. Low Muscle Mass

Muscle mass was derived from computed tomography (CT) images taken for routine diagnostic and staging purposes using the Alberta Protocol [29]. Muscle cross-sectional area (cm^2^) was measured by one researcher (NK) using a single cross-sectional axial image at the third lumbar vertebrae (L3) using SliceOmatic software (version 5.0, Tomovision, Magog, QC, Canada). Total muscle cross-sectional area (cm^2^) was determined within a HU range of −29 to +150 and was normalised for height (m^2^) to determine skeletal muscle index (cm^2^/m^2^). Low skeletal muscle index (termed low muscle mass herein) was defined as <43 cm^2^/m^2^ in men with a body mass index (BMI) < 24.9 kg/m^2^; <53 cm^2^/m^2^ in men with a BMI ≥ 25 kg/m^2^; and <41cm^2^/m^2^ in women of any BMI [30].

### 2.7. Sarcopenia

Sarcopenia was assessed according to the revised definition by the European Working Group for Sarcopenia in Older People (EWGSOP2) [6], including low muscle strength (probable sarcopenia), low muscle mass (sarcopenia) and poor physical performance (severe sarcopenia). Muscle strength was assessed using digital hand dynamometry (Jamar Digital Plus) per the American Society of Hand Therapists protocol [31]. Low muscle strength was defined as maximum grip strength < 27 kg (male) or <16 kg (female) from three assessments on each hand. Low muscle mass was defined as previously described. Physical performance was assessed using the Short Physical Performance Battery, a composite test that assesses gait speed, balance, and a chair-stand test [32]. Low physical performance was defined as a total score ≤ 8 (maximum score of 12).

### 2.8. Demographic Information and Potential Confounders

Baseline demographic data included age, sex, living situation (alone or with others), level of education (none, primary, secondary, or tertiary/university) and smoking status (never smoked, current smoker, ex-smoker). Disease type (NSCLC or SCLC), stage (I to IV for NSCLS; limited or extensive for SCLC), treatment plan (radiotherapy ± concurrent chemotherapy) and comorbidities (Charlson Comorbidity Index score) were retrieved from electronic medical records. Physical activity was assessed via the International Physical Activity Questionnaire (IPAQ), with activity levels classified as high (≥3000 MET-min/week), moderate (≥600 to <3000 MET-min/week), or low (<600 MET-min/week). BMI was estimated from body weight (standard floor scales; Tanita InnerScan 50, Tanita Australia) and height (self-reported or from medical records) and categorised as underweight (<18.5 kg/m^2^), healthy (18.5–24.9 kg/m^2^), overweight (BMI ≥ 25 kg/m^2^), or obese (BMI ≥ 30 kg/m^2^) according to WHO definitions [33].

### 2.9. Statistical Analysis

Descriptive statistics were reported as mean ± SD (continuous) and count (%) (categorical). Exposure variables were standardised a priori dietary pattern adherence scores (z-scores; continuous). Dietary pattern adherence scores were categorised into tertiles for descriptive comparisons of energy and nutrient intakes across tertiles of adherence. Outcome variables were malnutrition (binary), low muscle mass (binary) and sarcopenia (binary). PG-SGA B (mild to moderate malnutrition) and C (severe malnutrition), and probable sarcopenia, sarcopenia and severe sarcopenia were combined to form binary variables herein termed ‘malnutrition’ and ‘(probable)-sarcopenia’, respectively.

Univariate and multivariate logistic regression models, odds ratios (OR) and 95% confidence intervals (CI) examined associations between a priori dietary patterns adherence scores (continuous, independent variables) and malnutrition, low muscle mass and (probable)-sarcopenia (binary, dependant variables). Potential confounding variables were identified prior to analysis using a directed acyclic graph which represents the researchers’ hypothesised confounders, covariates and mediators of the relationship between dietary patterns and outcomes (Figure S1). Due to the limited sample size of this study, a pragmatic approach was taken to select the two most statistically relevant covariates for inclusion in the multivariate model. Bivariate analysis was used to individually assess associations between each potential confounder and the outcomes; the two covariates with the strongest evidence of a relationship with the outcomes (lowest *p*-value for the association) were included in the final multivariate models (Tables S3 and S4). This approach was utilised to include the most important (data-based) covariates while avoiding overfitting the data and allowed for a different set of covariates for the three outcomes. Due to the limited number of cases of (probable)-sarcopenia, only unadjusted analyses were conducted. Treatment-related factors were not examined (i.e., treatment type or dose) as this study used cross-sectional data collected prior to or at the commencement of cancer treatment.

To assess potential demographic differences between participants included in the analysis (*n* = 47) and those excluded for not completing the three-day food diary (*n* = 14), two-sided *t*-tests were used for continuous variables, and Fisher’s exact tests were used for categorical variables.

Independent sample *t*-tests were conducted to compare the mean a priori dietary pattern adherence scores (DGI-2013 and MDS) of participants with malnutrition, low muscle mass or (probable)-sarcopenia and those without the conditions. Mean difference and 95% CIs were reported. Participants with missing data for exposures or outcomes were excluded. All statistical analyses were performed using Stata/MP version 17 (Stata Corp., College Station, TX, USA). Statistical significance was considered at *p* < 0.05.

No power calculations were conducted for this secondary data analysis. A power calculation was conducted to determine a recruitment target of 120 participants for the original PREDICT study. Calculations were based on the strength of associations between individual predictors of muscle loss and outcomes in the population that could be detected with 80% power, given the expected size of analysis samples. Full details of the power analysis are reported elsewhere [16]. A post hoc power calculation for the present study (*n* = 47) indicated that a moderate effect size (odds ratio 0.4 [34]) for the association between standardised continuous independent variables and malnutrition (36.2% malnourished) could be detected with 72% power (α = 0.05).

## 3. Results

Overall, 976 patients were screened for eligibility and 154 met the eligibility criteria. Of these, 29 patients could not be approached for various reasons including an inability to collect baseline data prior to or within 5 days of commencing treatment. Sixty-eight patients were recruited, seven (10%) were not included due to changes in eligibility status (change in treatment protocol), and 14 (20.6%) did not complete baseline food records. Therefore, 47 participants were included in the present study (Figure S2). Due to missing CT data, only 44 participants were assessed for low muscle mass and (probable)-sarcopenia (where low muscle mass is a key criterion).

Table 1 presents the baseline characteristics of all participants, overall and by sex. Mean ± SD age was 70.6 ± 8.6 years, and 57.5% of participants were male. Most participants were diagnosed with NSCLC (91.5%). Stage IIIA disease (44.7%) and chemo-radiotherapy (66%) were the most common disease stage and treatment modality, respectively. The prevalence of malnutrition was 36.2%, low muscle mass was 50.0% and (probable)-sarcopenia was 13.6%. Characteristics were not statistically different between the included participants and those who were excluded for non-completion of the food record (Table S5).

### 3.1. Dietary Guideline Index 2013

A summary of DGI-2013 scores, as well as the percentage of people achieving DGI-2013 recommendations are summarised in Table S6. The mean ± SD total DGI-2013 score was 53.0 ± 13.0 and was comparable between men (53.0 ± 14.0) and women (53.1 ± 11.7). Participants scored highest on the component ‘limit intake of alcohol’ (9.36 ± 2.47), indicating high compliance. Participants scored lowest on ‘consume mostly low-fat milk’ (0.47 ± 1.48) and ‘total beverage intake’ (1.02 ± 0.76), indicating poor compliance. Few participants met the guidelines for vegetables (15%), fruits (4%), cereals (2%), meat and alternatives (17%) or dairy and alternatives (0%) or their sub-components. No (0%) participants achieved a full score for food variety. However, 60% of participants met the requirement to limit discretionary foods. Participants in the highest tertile of DGI-2013 adherence consumed the lowest mean total energy (kJ) but highest percentage energy (%E) of protein and dietary fibre (g/day), compared to tertiles 1 and 2 (Table 2).

Mean DGI-2013 adherence scores were not significantly different between those with and without malnutrition (mean difference: 5.77; 95% CI: −2.06, 13.60), low muscle mass (mean difference: 0.87; 95% CI: −6.83, 8.56) and (probable)-sarcopenia (mean difference: 3.67; 95% CI: −7.34, 14.69).

### 3.2. Mediterranean Diet Score

A summary of the MDSs, as well as the percentage of participants meeting MDS recommendations, is displayed in Table S7. The mean ± SD total MDS was 3.6 ± 1.5, which was similar for men (3.5 ± 1.5) and women (3.7 ± 1.7). The highest average MDS component scores were for fruits and nuts, cereals and grains, mono-polyunsaturated fat ratio, and meat and meat products (all 0.51 ± 0.51), indicating higher compliance compared to other foods. The lowest average MDS component score was for legumes (0.06 ± 0.25). Few participants met the recommendations for legumes (6%), alcohol (21%) or fish and seafood (26%). All other MDS recommendations were achieved by 50% of the participants. The highest tertile of MDS adherence consumed the lowest mean total energy (kJ), %E protein %E total and saturated fats, and %E total sugars, compared to tertiles 1 and 2 (Table 2).

There were no statistically significant differences between mean MDS in participants with and without malnutrition (mean difference: 0.35; 95% CI: −0.60, 1.30); low muscle mass (mean difference: 0.14; 95% CI: −0.78, 1.05) and (probable)-sarcopenia (mean difference: −0.87; 95% CI: −2.17, 0.43).

### 3.3. Dietary Patterns and Odds of Malnutrition, Low Muscle Mass and (Probable)-Sarcopenia

As shown in Table 3, a priori dietary pattern scores were not significantly associated with odds of malnutrition, low muscle mass or (probable)-sarcopenia.

Models adjusted for: physical activity (MET-min/week) and comorbidities (malnutrition); sex (male or female) and comorbidities (low muscle mass). Due to a small number of cases of (probable)-sarcopenia, an adjusted model is not presented to avoid over fitting.

## 4. Discussion

This study of 47 men and women prior to or at the commencement of (chemo)radiotherapy for lung cancer found that patients exhibited poor diet quality as indicated by sub-optimal adherence to the DGI-2013 and MDS, a priori dietary patterns which estimate adherence to the Australian Dietary Guidelines and the Mediterranean diet, respectively. One-third (36.2%) of patients were malnourished, half (50.0%) had low muscle mass and 13.6% had (probable)-sarcopenia. However, adherence to the DGI-2013 or MDS was not associated with these outcomes. Therefore, it remains uncertain whether diet quality is important during cancer treatment, especially in terms of its influence on nutritional status and muscle-related outcomes.

Few studies have examined the associations between diet quality and nutritional status among adults with cancer [35]. Whilst the present study showed that diet quality was not associated with malnutrition, low muscle mass or (probable)-sarcopenia, findings among healthy older adults are conflicting. Some observational studies have observed similar non-significant associations between diet quality and nutritional status in healthy older adults [36], while a cross-sectional analysis of community-dwelling older adults in Spain found that adherence to high-quality a priori dietary patterns including the Mediterranean diet (Mediterranean Diet Screener) or Alternative Healthy Eating Index were associated with lower prevalence of malnutrition, independent of energy and protein intake [37]. There are likely multiple reasons for these conflicting findings, including the unique nutritional challenges and higher energy and protein requirements of people with cancer. Whilst the present analysis found those with the highest adherence to DGI-2013 consumed the highest percentage of energy from protein (21.9%) compared to tertiles 1 and 2, this may be insufficient to support muscle health among people with cancer, where the requirement for intake up to 2 g/kg has been suggested [38]. Moreover, there were different a priori dietary patterns assessed between studies. The small sample size and cross-sectional design of the present study also need to be considered with caution. As such, further large-scale studies are warranted to explore the link between diet quality and nutrition- and muscle-related outcomes in people with cancer.

People with cancer are encouraged to consume diets rich in energy and protein, in line with disease-specific guidelines [7]. In practice, the food-based strategies reportedly used by dietitians to achieve these nutrient recommendations vary substantially [39]. A recent qualitative study of Australian dietitians revealed differing opinions on whether to prioritise overall health and long-term well-being through high-quality dietary intake, or to focus solely on meeting protein and energy needs, including with ‘discretionary’ foods [39]. Whilst the present analysis did not find an association between diet quality and malnutrition, low muscle mass or (probable)-sarcopenia, further research into these potential associations may inform the clinical decision making of dietitians.

The Mediterranean diet has numerous demonstrated benefits for people with cancer, including improved quality of life and reduced cancer-related fatigue [12]. A systematic review published in 2024 found the Mediterranean diet to be both feasible and safe for adults with cancer but emphasised the lack of evidence regarding its effects on outcomes beyond weight loss [40]. Moreover, in a pilot randomised control trial of 23 men with prostate cancer, lean body mass was reduced (−1.5 kg) after 8 weeks of Mediterranean diet compared to usual care [12]. Thus, it has previously been postulated that any negative impacts of the Mediterranean diet on muscle mass are, at least in part, attributable to the limited energy and protein content of this dietary pattern combined with the heightened nutritional needs of people with cancer [12]. In accordance, our findings demonstrate that those with the highest diet quality consumed lower total energy (MDS and DGI-2013) and protein (% energy, MDS only) compared to those with the poorest adherence. This lower energy intake among those with the highest adherence to MDS and DGI-2013 may offset other potential benefits of a high-quality dietary pattern. Future Mediterranean diet interventions, including high energy and protein content, may be an alternative strategy to improve cancer-related outcomes including nutritional status and muscle mass using a high-quality diet approach.

### Potential Limitations

The present study provides novel insights into the role of a priori dietary patterns for nutrition- and muscle-related outcomes in people with lung cancer. However, the findings should be considered in light of several limitations. Three-day food diaries, as used in this analysis, provide a detailed understanding of current dietary intake. However, they may be subject to under-reporting and reactivity bias, where participants adapt their dietary intake under observation. Three-day food diaries may not accurately capture habitual dietary intake due to day-to-day dietary variability related to symptoms or treatment effects. As such dietary findings should be considered in light of these potential limitations. Estimation of DGI-2013 and MDS adherence may also be associated with limitations. Of note, mixed dishes (e.g., stir-fry) were not disaggregated, which may have under-estimated the intake of some food groups. Two components of the DGI-2013 (added salt and trimming of meat) were not captured in the dietary assessment and were therefore excluded from the analysis. Further, if an item was not reported, it was assumed to not have been consumed, which may underestimate consumption of frequently forgotten foods (e.g., water, confectionery, tea, and coffee). A key limitation of the MDS is its reliance on sample medians (i.e., median food group intake in grams), and therefore, adherence scores are dependent on habitual dietary characteristics of the studied population and may impact generalizability. The small sample size is a key limitation, with reasonable power (>70%) to detect moderate effect sizes, but low power for detecting small, but potentially clinically important, effects. Sample size also affected our ability to adjust regression models for all pre-identified potential confounders, such as energy intake which was found to be lowest in the highest tertiles of DGI-2013 and MDS adherence (Table 2). The included sample was diverse in cancer type (NSCLC and SCLC) and stage, and further research should examine whether associations differ across these groups. Moreover, a proportion of eligible patients did not complete the three-day food records and were therefore excluded from the analysis (Table S2). This may have introduced selection bias, as patients who were unable to complete the food diary (e.g., due to illness, time burden or insufficient literacy levels) may be under-represented. This could affect the observed prevalence of outcomes and generalisability of the results. However, those excluded from the analyses were not statistically different from those in the included sample. Finally, the cross-sectional study design precludes causal inference and reverse causality where participants may adapt their dietary intake due to the presence of malnutrition, low muscle mass or (probable)-sarcopenia. The study design also limits the ability to examine potential long-term prospective associations between diet quality and the outcomes of interest.

## 5. Conclusions

People with lung cancer prior to or at the commencement of treatment had low diet quality, as measured by adherence to the Australian Dietary Guidelines (DGI-2013) and Mediterranean diet (MDS), but diet quality was not associated with odds of having malnutrition, low muscle mass or (probable)-sarcopenia in this sample. As such, it remains unclear whether diet quality is important during lung cancer treatment in relation to its impact on nutritional status and muscle-related outcomes. Large-scale, prospective dietary studies before and during treatment are needed to elucidate whether diet quality has a role in the nutritional management of people with cancer.

## Figures and Tables

**Table 1:** Baseline characteristics of patients with lung cancer undergoing treatment, overall and by sex.

Characteristics	Total	Male	Female
N (%)	47 (100)	27 (57.5)	20 (42.5)
Age (years), mean ± SD	70.6 ± 8.6	72.4 ± 8.9	68.2 ± 7.9
Cancer type, n (%)			
NSCLC	43 (91.5)	26 (96.3)	17 (85.0)
SCLC	4 (8.5)	1 (3.7)	3 (15.0)
Disease stage, n (%)			
IA	2 (4.3)	2 (7.4)	0 (0.0)
IB	1 (2.1)	1 (3.7)	0 (0.0)
IIA	2 (4.3)	1 (3.7)	1 (5.0)
IIB	8 (17.0)	4 (14.8)	4 (20.0)
IIIA	21 (44.7)	14 (51.9)	7 (35.0)
IIIB	7 (14.9)	2 (7.4)	5 (25.0)
IV	3 (6.4)	2 (7.4)	1 (5.0)
Limited	3 (6.4)	1 (3.7)	2 (10.0)
Treatment type, n (%)			
Radiotherapy	16 (34.0)	11 (40.7)	5 (25.0)
Chemo-radiotherapy	31 (66.0)	16 (59.3)	15 (75.0)
Smoking status, n (%)			
Never smoked	4 (8.5)	1 (3.70)	3 (15.00)
Current smoker	9 (19.2)	4 (14.81)	5 (25.00)
Ex-smoker	34 (72.3)	22 (81.48)	12 (60.00)
Charlson co-morbidity index, mean ± SD	1.9 ± 1.7	2.1 ± 1.8	1.5 ± 1.5
Living situation, n (%)			
Alone	10 (21.3)	5 (18.5)	5 (25.0)
With others	37 (78.7)	22 (81.5)	15 (75.0)
Highest level of education, n (%)			
Primary	7 (15.2)	5 (19.2)	2 (10.0)
Secondary	31 (67.4)	16 (61.5)	15 (75.0)
Tertiary/university	8 (17.4)	5 (19.2)	3 (15.0)
Physical activity level, n (%) ^1^			
Low	26 (55.3)	11 (40.7)	15 (75.0)
Moderate	12 (25.5)	8 (29.6)	4 (20.0)
High	9 (19.2)	8 (29.6)	1 (5.0)
BMI (kg/m^2^), n (%) ^2^			
Underweight	5 (10.6)	4 (14.8)	1 (5.0)
Healthy	14 (29.8)	6 (22.2)	8 (40.0)
Overweight	17 (36.2)	14 (51.9)	3 (15.0)
Obesity	11 (23.4)	3 (11.1)	8 (40.0)
Energy intake (kJ), mean ± SD	7637 ± 2295	8180 ± 2189	6903 ± 2283
Malnutrition, n (%) ^3^			
Well nourished	30 (63.8)	19 (70.4)	11 (55.0)
Malnourished	17 (36.2)	8 (29.6)	9 (45.0)
Mild-moderately malnourished	16 (34.0)	7 (25.9)	9 (45.0)
Severely malnourished	1 (2.1)	1 (3.7)	0 (0.0)
Muscle mass, n (%) ^4^			
Normal	22 (50.0)	10 (41.7)	12 (60.0)
Low	22 (50.0)	14 (58.3)	8 (40.0)
(Probable)-sarcopenia, n (%) ^5^			
Non-sarcopenic	38 (86.4)	20 (83.3)	18 (90.0)
Sarcopenic	6 (13.6)	4 (16.7)	2 (10.0)
Probable sarcopenia	1 (2.2)	0 (0.0)	1 (5.0)
Sarcopenia	4 (8.9)	3 (12.0)	1 (5.0)
Severe sarcopenia	1 (2.2)	1 (4.0)	0 (0.0)

Abbreviations: BMI, body mass index; NSCLC, non-small cell lung cancer; SCLC, small cell lung cancer. ^1^ Physical activity level determined using the International Physical Activity Short Form (IPAQ): low < 600 MET-min/week; moderate ≥ 600–300 MET-min/week; high ≥ 3000 MET-min/week. ^2^ BMI: underweight (<18.5 kg/m^2^); healthy (18.5–24.9 kg/m^2^); overweight (25–29.9 kg/m^2^); obese (>30 kg/m^2^). ^3^ Malnutrition diagnosed using Patient Generated Subjective Global Assessment (PG-SGA): mild-moderate malnutrition (PG-SGA B); severe malnutrition (PG-SGA C). ^4^ Muscle mass assessed using diagnostic computed tomography (CT) scans at the third lumbar vertebrae: low muscle mass (<43 cm^2^/m^2^ in men with a body mass index < 24.9 kg/m^2^; <53 cm^2^/m^2^ in men with a body mass index ≥ 25 kg/m^2^; and <41 cm^2^/m^2^ in women). *N* = 44 due to missing data. ^5^ (Probable)-sarcopenia diagnosed according to revised European Working Group on Sarcopenia in Older People 2019 definition (EWGSOP2): probable sarcopenia (handgrip strength < 27 kg (male) and <16 kg (female)); sarcopenia (low handgrip strength plus low muscle mass); severe sarcopenia (low handgrip strength plus low muscle mass plus Short Physical Performance Battery (SPPB) score ≤ 8). *N* = 44 due to missing data.

**Table 2:** Energy and nutrient intakes across tertiles of dietary pattern scores in men and women with lung cancer prior to or at commencement of treatment (*n* = 47).

	Dietary Pattern Scores		
Dietary Guideline Index 2013			
	Tertile 1 (*n* = 16)	Tertile 2 (*n* = 16)	Tertile 3 (*n* = 15)
Adherence score, mean ± SD	38.2 ± 6.9	54.5 ± 2.5	67.2 ± 5.1
Nutrients			
Total energy (kJ/day)	9021 ± 2597	7236 ± 2036	6691 ± 1563
Protein (% energy)	16.5 ± 3.1	20.6 ± 3.8	21.9 ± 5.0
Total fat (% energy)	38.1 ± 4.8	34.3 ± 5.6	35.7 ± 7.4
Saturated fat (% energy)	15.4 ± 3.5	13.5 ± 2.8	13.7 ± 0.7
Carbohydrate (% energy)	41.6 ± 7.8	43.3 ± 8.2	40.9 ± 9.8
Total sugars (% energy)	18.1 ± 6.5	18.4 ± 7.5	17.8 ± 8.2
Dietary fibre (g/day)	20.4 ± 6.7	19.1 ± 8.1	21.4 ± 8.3
Mediterranean Diet Score			
	Tertile 1 (*n* = 25)	Tertile 2 (*n* = 9)	Tertile 3 (*n* = 13)
Adherence score, mean ± SD	2.4 ± 0.8	4.0 ± 0.0	5.5 ± 0.8
Nutrients			
Total energy (kJ/day)	7941 ± 2751	7921 ± 1066	6973 ± 1929
Protein (% energy)	19.4 ± 4.0	20.7 ± 6.0	19.2 ± 4.8
Total fat (% energy)	36.2 ± 5.8	36.9 ± 7.9	35.12 ± 5.6
Saturated fat (% energy)	15.2 ± 2.8	14.3 ± 3.8	12.2 ± 3.5
Carbohydrate (% energy)	42.3 ± 8.4	40.9 ± 10.3	42.1 ± 8.0
Total sugars (% energy)	18.8 ± 7.9	18.7 ± 8.4	16.5 ± 5.1
Dietary fibre (g/day)	18.7 ± 7.1	24.0 ± 9.1	20.9 ± 7.0

**Table 3:** Odds ratios (OR) with 95% confidence intervals (CI) for associations between dietary patterns and malnutrition (*n* = 47), low muscle mass and (probable)-sarcopenia in men and women with lung cancer prior to or at commencement of treatment (*n* = 44).

		OR (95% CI)	
Dietary Patterns	Malnutrition ^1^	Low Muscle Mass ^2^	(Probable)-Sarcopenia ^3^
Dietary Guideline Index 2013			
Univariate	0.63 (0.34, 1.18)	0.93 (0.50, 1.73)	0.73 (0.29, 1.80)
Multivariate	0.67 (0.35, 1.28)	0.90 (0.47, 1.70)	-
Mediterranean Diet Score			
Univariate	0.79 (0.43, 1.46)	0.91 (0.49, 1.69)	1.82 (0.72, 4.85)
Multivariate	0.75 (0.37, 1.49)	0.98 (0.51, 1.88)	-

Abbreviations: CI, confidence interval; OR, odds ratio. ^1^ Malnutrition diagnosed using Patient Generated Subjective Global Assessment (PG-SGA), *n* = 47. ^2^ Muscle mass assessed using diagnostic computed tomography (CT) scans at the third lumbar vertebrae: low muscle mass (<43 cm^2^/m^2^ in men with a body mass index < 24.9 kg/m^2^; <53 cm^2^/m^2^ in men with a body mass index ≥ 25 kg/m^2^; and <41 cm^2^/m^2^ in women), *n* = 44 due to missing data. ^3^ (Probable)-sarcopenia diagnosed according to revised European Working Group on Sarcopenia in Older People 2019 definition (EWGSOP2): probable sarcopenia (handgrip strength < 27 kg (male) and <16 kg (female)); sarcopenia (low handgrip strength plus low muscle mass); and severe sarcopenia (low handgrip strength plus low muscle mass plus Short Physical Performance Battery (SPPB) score ≤ 8), *n* = 44 due to missing data.

## Data Availability

The dataset presented in this article is not readily available. This project received ethics approval from Peter MacCallum Cancer Centre to conduct this study, and approval does not extend to publicly sharing data beyond the research team who meet the criteria for access to personal data. Requests to access the datasets should be directed to the corresponding author.

## Supplementary Materials

The following supporting information can be downloaded at: https://www.mdpi.com/article/10.3390/nu18050764/s1, Table S1: Components and scoring methods for the Dietary Guideline Index 2013; Table S2: Components and scoring methods for the Mediterranean Diet Score; Table S3: Potential confounders associated with malnutrition in people with lung cancer; Table S4: Potential confounders associated with low muscle mass in people with lung cancer; Table S5: Baseline characteristics of patients with lung cancer undergoing treatment in the included (*n* = 47) and excluded (*n* = 14) samples; Table S6: Dietary Guideline Index 2013 percentage of non-consumers, component scores and percentage of participants meeting dietary guidelines, overall and by sex; Table S7: Mediterranean Diet Score percentage of non-consumers, component scores and percentage of participants meeting dietary guidelines, overall and by sex; Figure S1: Directed Acyclic Graph; Figure S2: Flow diagram of eligible participants from the PREDICT study.

## Abbreviations

The following abbreviations are used in this manuscript:

ASA24	Automated Self-Administered 24-hour
AUSNUT	Australian Food, Supplement, and Nutrient Database
CI	confidence intervals
CT	computed tomography
DGI 2013	Dietary Guideline Index 2013
EWGSOP	European Working Group for Sarcopenia in Older People
IPAQ	International Physical Activity Questionnaire
L3	third lumbar vertebrae
MDS	Mediterranean Diet Score
NSCLC	non-small cell lung cancer
OR	odds ratios
PG-SGA	Patient Generated Subjective Global Assessment
PREDICT	Predicting Muscle Loss During Lung Cancer Treatment
SCLC	Small cell lung cancer h
