# Supplementary material for: Diet Quality Is Not Associated with Malnutrition, Low Muscle Mass and Sarcopenia During Lung Cancer Treatment: A Cross-Sectional Study
Source: Nutrients. 2026 Feb 26;18(5):764. doi: 10.3390/nu18050764 (PMC12986464; doi:10.3390/nu18050764)
Supplement: Supplementary file 1 [file nutrients-18-00764-s001.zip › Figure S1.pdf]

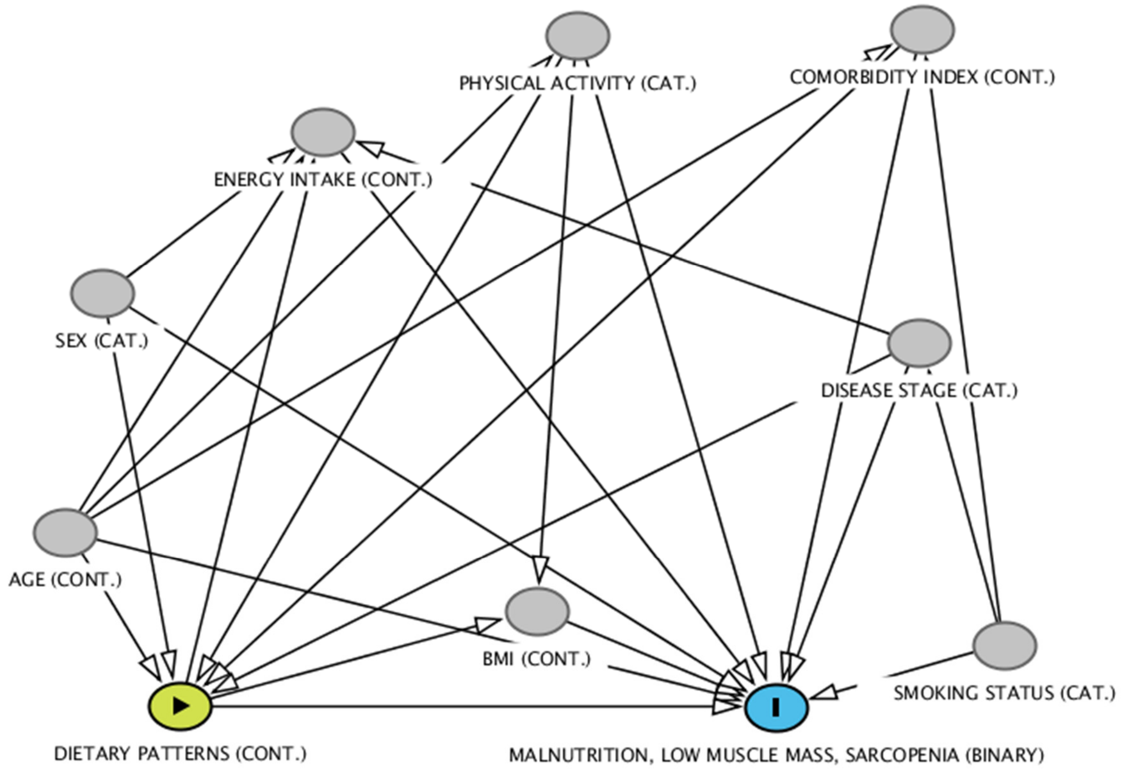

Supplementary Figure S1 *Directed Acyclic Graph of proposed relationships between dietary patterns (exposure) and malnutrition, low muscle mass and (probable)sarcopenia (outcomes). This figure includes the casual pathways as well as potential confounders (age, sex, energy intake, physical activity level, comorbidities index, and disease stage), covariates (smoking status), and mediators (body mass index)*
