# Supplementary material for: Diet Quality Is Not Associated with Malnutrition, Low Muscle Mass and Sarcopenia During Lung Cancer Treatment: A Cross-Sectional Study
Source: Nutrients. 2026 Feb 26;18(5):764. doi: 10.3390/nu18050764 (PMC12986464; doi:10.3390/nu18050764)
Supplement: Supplementary file 1 [file nutrients-18-00764-s001.zip › Table S1.pdf]

Supplementary Table S1. Components and scoring methods for the Dietary Guideline Index 2013

Dietary Guideline	Description*	Criteria for maximum score	Minimum – maximum scores
**Guidelines for adequate intake**			
1. Enjoy a wide variety of nutritious foods	**Food variety:** > 15 g/day (beverages) and > 20 g/day (foods) from five food groups	100%	0 – 10 (proportional score)
2. Enjoy plenty of vegetables	**Total vegetable intake:** servings of vegetables per day (75g/serve)	M ≥ 6, F ≥ 5 (19-50 years)<br>M ≥ 5.5, F ≥ 5 (51-70 years)<br>M ≥ 5, F ≥ 5 (> 70 years)	0 – 10 (proportional score)
3. Enjoy fruit	**Total fruit intake:** servings of fruit per day (150g/serve)	≥ 2	0 – 10 (proportional score)
4. Enjoy grain (cereal) foods, mostly wholegrain or high cereal fibre varieties	**Total cereals intake:** servings of cereals per day (500kJ/serve)	M ≥ 6, F ≥ 6 (19-50 years)<br>M ≥ 6, F ≥ 4 (51-70 years)<br>M ≥4.5, F ≥ 3 (> 70 years)	0 – 5 (proportional score)
	**Mostly wholegrain:** proportion of wholegrain bread to total bread intake per day	≥50% wholegrain bread	0 – 5 (proportional score)
5. Enjoy lean meats, poultry, fish and alternatives	**Total meat and alternatives intake:** servings of meat and alternatives per day (550kJ/serve)	M ≥ 3, F ≥ 2.5 (19-50 years)<br>M ≥ 2.5, F ≥ 2 (51-70 years)<br>M ≥2.5, F ≥ 2 (> 70 years)	0 – 5 (proportional score)
	**Lean meat:** proportion of lean meat and alternatives to total meat and alternative intake per day	100% lean meat	0 – 5 (proportional score)

6. Enjoy milk, yoghurt, cheese and alternatives, mostly reduced fat	**Total dairy and alternatives intake:** servings of dairy and alternatives per day (550kJ/serve)	M ≥ 2.5, F ≥ 2.5 (19-50 years)<br>M ≥ 2.5, F ≥ 4 (51-70 years)<br>M ≥3.5, F ≥ 4 (> 70 years)	0 – 10 (proportional score)
7. Drink plenty of water	**Total beverages intake:** servings of beverages per day (250mL/serve)	M ≥ 10, F ≥ 8 – 0	0 – 5 (proportional score)
	**Mostly water:** proportion of water to total beverages intake per day	≥50% water intake	0 – 5 (proportional score)
**Guidelines to limit or moderate intake**			
8. Limit intake of foods containing saturated fat, added salt, added sugars and alcohol	**Limit discretionary choices:** servings of discretionary foods per day (600kJ/serve)	M ≤ 3; F ≤ 2.5 - M > 3; F > 2.5	0 – 10 (binary score)
9. Limit intake of food high in saturated fat	**Choose reduced-fat milk:** proportion of reduced-fat milk to total milk intake per day	≥50% Reduced fat milk	0 – 5 (binary score)
10. Small allowance of unsaturated fats, oils and spreads	**Unsaturated fats intake:** servings of unsaturated fats per day (250kJ/day)	[M ≤ 4, F ≤ 2 (19-50 and 51-70 years)<br>M ≤ 2, F ≤ 2 (> 70 years)] –<br>M > 4, F > 2 2	0 – 10 (binary score)
12. Limit intake of foods and drinks containing added sugars	**Limit added sugar intake:** servings of added sugar per day (600kJ/serve)	M ≤ 1.5; F ≤ 1.25 – M > 1.5; F > 1.25	0 – 10 (binary score)
13. Limit intake of alcohol	**Limit alcohol intake:** servings of alcohol per day (600kJ/serve)	≤ 2 - > 2	0 – 10 (binary score)

***Abbreviations**: M, male; F, female *Serve sizes according to the Australian Dietary Guidelines, reported as g or kJ. Where serve size was reported as a range, the mid-point was selected.*
