# Supplementary material for: Diet Quality Is Not Associated with Malnutrition, Low Muscle Mass and Sarcopenia During Lung Cancer Treatment: A Cross-Sectional Study
Source: Nutrients. 2026 Feb 26;18(5):764. doi: 10.3390/nu18050764 (PMC12986464; doi:10.3390/nu18050764)
Supplement: Supplementary file 1 [file nutrients-18-00764-s001.zip › Table S2.pdf]

Supplementary Table S2. Components and scoring methods for the Mediterranean Diet Score

Dietary component	Included foods	Scoring criteria
1. Vegetables	Potatoes, cabbage/cauliflower/brassica vegetables (cabbage, broccoli, broccolini and cauliflower), carrots (including other root vegetables), leaf and stake vegetables (leaf, vegetables, stalk vegetables, fresh herbs, seaweeds), peas and beans (peas, beans, sprouts), tomato, other fruiting vegetables (pumpkin, squash, mushroom, sweetcorn), other vegetables.	Sex-specific median intake cut points.<br>Above or equal to median score = 1<br>Below median score = 0<br>(Components 1 – 6)
2. Legumes	Legumes, pulses, and dips (legume)	
3. Fruits and nuts	Pome fruits (apples and pears), berry fruits, citrus fruits (oranges, lemons, limes, and other citrus fruits), stone fruits (peaches, nectarines, and other stone fruits), tropical and subtropical fruits (bananas, pineapples, and other tropical and sub-tropical fruits), other fruits, dried/preserved fruits (vine-dried fruits, other dried fruits including fruit mixes), dried fruit and nut mixes, and preserved fruits, whole seeds and nuts (peanuts, coconuts, other nuts, mixed nuts)	
4. Cereals and grains	Pasta and noodles (wheat based), instant noodles and noodle products (wheat based), pasta and noodles (not wheat based), filled pasta, grains (other than rice) and grain fractions, rice and rice grain fractions, cereal flours and starches (including fortified), bread and bread rolls (white, mixed grain, wholemeal and brown, rye, gluten free or other), English-style muffins, flat breads (wheat and other cereal flours), savoury filled or topped breads and bread rolls, sweet bread, buns and scrolls (unfilled and filled), fried bread products, breakfast cereal (corn, rice or wheat based, mixed grain, with fruit and/or nuts) and porridge style (oat based or other cereals)	
5. Fish and seafood	White fin fish, crustacea and molluscs (fresh, frozen, wild caught and packed, battered or crumbed) and smoked fish, oily fish (i.e., salmon, tinned salmon, herring, mackerel, sardines or tuna steak), tinned tuna, fish row and eel	
6. Mono-unsaturated fat: saturated fat ratio	Ratio of monounsaturated fats to saturated fats	

7. Dairy products	Cow milks (regular, reduced fat or skimmed), evaporated milks, milk powders, non-bovine species milk, soy-based beverages (regular, reduced fat or skim; fortified or non-fortified), milk-based drink (coffee – latte/cappuccino, chocolate or other flavoured; regular or reduced fat), milk-based fruit drinks and flavoured soy-based beverages (regular or reduced fat) and breakfast cereal beverages, hard cheese (ripened or unripened; regular or reduced fat), soft cheese (camembert, brie and other surface ripened cheeses), processed cheese (regular and reduced fat), and cheese substitutes, yoghurt (natural or flavoured; regular, reduced fat or skimmed), yoghurt drinks (buttermilk), soy-based yoghurts (regular or reduced fat), and dips (dairy-based).	Sex-specific median intake cut points.<br>Below median score = 1<br>Above or equal to median score = 0<br>(Components 7 – 8)
8. Meat and meat products	Sausages, Frankfurt's and saveloys (regular and reduced fat), bacon, ham, fermented meats (salami), processed delicatessen meats (mammalian or poultry), canned meats, dried meats, beef, lamb or mutton, pork, veal, other mammalian game (kangaroo) and dishes, meat substitutes and dishes and organ meats/offal (liver, kidney, other organ meats and offal, liver paste/pate and dishes), chicken, other poultry, feather game, and dishes/soups, eggs (chicken and other)	
9. Alcohol	Wine, beer, cider and perry, spirits and other alcoholic beverages	No more than 2 serves/day = 1<br>No alcohol or > 2 serves per day = 0
