# Supplementary material for: Diet Quality Is Not Associated with Malnutrition, Low Muscle Mass and Sarcopenia During Lung Cancer Treatment: A Cross-Sectional Study
Source: Nutrients. 2026 Feb 26;18(5):764. doi: 10.3390/nu18050764 (PMC12986464; doi:10.3390/nu18050764)
Supplement: Supplementary file 1 [file nutrients-18-00764-s001.zip › Table S3.pdf]

**Supplementary Table S3.** Potential confounders associated with malnutrition in people with lung cancer (n = 47)

| Potential confounder | OR (95% CI) | P-value | Inclusion in analysis |
|---|---|---|---|
| Age (years) | 0.98 (0.91, 1.05) | 0.51 | NO |
| Sex | 1.94 (0.58, 6.50) | 0.28 | NO |
| Energy intake (kJ) | 1.00 (1.00, 1.00) | 0.96 | NO |
| Physical activity | 1.00 (1.00, 1.00) | **0.16** | **YES** |
| Comorbidity Index | 0.75 (0.49, 1.15) | **0.18** | **YES** |
| Disease stage[1] | 0.88 (0.07, 10.43) | 0.92 | NO |
| Smoking status[2] | 1.78 (0.17, 18.57) | 0.63 | NO |

*Univariate logistic regression; Odds Ratios (OR) and 95% confidence intervals (CI) for associations between potential confounding variables and malnutrition. Bolding indicates lowest p-value.*
*Age (years), energy intake (kJ), physical activity (MET-min/week) and Comorbidity Index score were continuous variables. Sex, disease stage and smoking status were binary variables.*
[1]*Disease stage: categorised into limited stage (stage IA, IB, IIA, IIB, IIIA, IIIB) and extensive stage (stage IV)*
[2]*Smoking status: categorised into never smoked and previous/current smoker.*
