# Supplementary material for: Diet Quality Is Not Associated with Malnutrition, Low Muscle Mass and Sarcopenia During Lung Cancer Treatment: A Cross-Sectional Study
Source: Nutrients. 2026 Feb 26;18(5):764. doi: 10.3390/nu18050764 (PMC12986464; doi:10.3390/nu18050764)
Supplement: Supplementary file 1 [file nutrients-18-00764-s001.zip › Table S4.pdf]

Supplementary Table S4. Potential confounders associated with low muscle mass in people with lung cancer (n = 44)

Potential confounder	OR (95% CI)	P-value	Inclusion in analysis
Age (years)	1.01 (0.94, 1.08)	0.86	NO
Sex	0.48 (0.14, 1.59)	**0.23**	**YES**
Energy intake (kJ)	1.00 (1.00, 1.00)	0.41	NO
Physical activity	1.00 (1.00, 1.00)	0.74	NO
Comorbidity Index	1.27 (0.87, 1.85)	**0.22**	**YES**
Disease stage[1]	2.10 (0.18, 25.00)	0.56	NO
Smoking status[2]	3.32 (0.32, 34.65)	0.32	NO

Univariate logistic regression; Odds Ratios (OR) and 95% confidence intervals (CI) for associations between potential confounding variables and low muscle mass. Bolding indicates lowest p-value. N=44 due to missing data.
Age (years), energy intake (kJ), physical activity (MET-min/week) and Comorbidity Index score were continuous variables. Sex, disease stage and smoking status were binary variables.
[1]Disease stage: categorised into limited stage (stage IA, IB, IIA, IIB, IIIA, IIIB) and extensive stage (stage IV)
[2]Smoking status: categorised into never smoked and previous/current smoker.
