# Supplementary material for: Diet Quality Is Not Associated with Malnutrition, Low Muscle Mass and Sarcopenia During Lung Cancer Treatment: A Cross-Sectional Study
Source: Nutrients. 2026 Feb 26;18(5):764. doi: 10.3390/nu18050764 (PMC12986464; doi:10.3390/nu18050764)
Supplement: Supplementary file 1 [file nutrients-18-00764-s001.zip › Table S5.pdf]

Table S5. Baseline characteristics of patients with lung cancer undergoing treatment in the included (n=47 and excluded (n=14) samples

**Characteristics**			
	**Included sample (n=47)**	**Excluded sample (n=14)**	**p-value**
**N (%)**	47 (100)	14 (100)	
**Age (years),** mean ± SD	70.6 ± 8.6	66.6 ± 12.1	0.18
**Sex**			
Female	27 (57.4)	11 (78.6)	0.21
Male	20 (42.6)	3 (21.4)	
**Cancer type,** n (%)			
NSCLC	43 (91.5)	11 (78.6)	0.34
SCLC	4 (8.5)	3 (21.4)	
**Disease stage,** n (%)			
IA	2 (4.3)	1 (7.1)	0.35
IB	1 (2.1)	0 (0.0)	
IIA	2 (4.3)	0 (0.0)	
IIB	8 (17.0)	2 (14.3)	
IIIA	21 (44.7)	3 (21.4)	
IIIB	7 (14.9)	4 (28.6)	
IV	3 (6.4)	0 (0.0)	
Limited	3 (6.4)	4 (28.6)	
**Treatment type,** n (%)			
Radiotherapy	16 (34.0)	3 (21.4)	0.52
Chemo-radiotherapy	31 (66.0)	11 (78.6)	
**Smoking status,** n (%)			
Never smoked	4 (8.5)	3 (21.4)	0.11
Current smoker	9 (19.2)	5 (35.7)	
Ex-smoker	34 (72.3)	6 (42.9)	
**Living situation,** n (%)			
Alone	10 (21.3)	1 (7.1)	0.43
With others	37 (78.7)	13 (92.9)	

**Highest level of education,** n (%)			
Primary	7 (15.2)	0 (0.0)	0.29
Secondary	31 (67.4)	10 (71.4)	
Tertiary/university	8 (17.4)	4 (28.6)	
**Physical activity level,** n (%)[1]			
Low	26 (55.3)	4 (28.6)	0.20
Moderate	12 (25.5)	5 (35.7)	
High	9 (19.2)	5 (35.7)	
**BMI (kg/m^2),** n (%)[2]			
Underweight	5 (10.6)	0 (0.0)	0.30
Healthy	14 (29.8)	5 (35.7)	
Overweight	17 (36.2)	8 (57.1)	
Obesity	11 (23.4)	1 (7.1)	
**Energy intake (kJ),** mean ± SD	7637 ± 2295	-	-
**Malnutrition,** n (%)[3]			
Well nourished	30 (63.8)	9 (69.2)	1.00
Malnourished	17 (36.2)	4 (30.8)	
**Muscle mass,** n (%)[4]			
Normal	22 (50.0)	7 (63.6)	0.51
Low	22 (50.0)	4 (36.4)	
**(Probable)-sarcopenia,** n (%)[5]			
Non-sarcopenic	38 (86.4)	10 (90.9)	0.58
Sarcopenic	6 (13.6)	1 (9.1)	

Abbreviations: *BMI, body mass index; NSCLC, non-small cell lung cancer; SCLC, small cell lung cancer.*

[1]Physical activity level determined using the International Physical Activity Short Form (IPAQ): low < 600 MET-min/week; moderate ≥600-300 MET-min/week; high ≥3000 MET-min/week.

[2]BMI: underweight (< 18.5 kg/m^2); healthy (18.5 – 24.9 kg/m^2); overweight (25 – 29.9 kg/m^2); obese (> 30 kg/m^2).

[3]Malnutrition diagnosed using Patient Generated Subjective Global Assessment (PG-SGA): mild-moderate malnutrition (PG-SGA B); severe malnutrition (PG-SGA C).

[4]Muscle mass assessed using diagnostic computed tomography (CT) scans at the third lumbar vertebrae: low muscle mass (< 43 cm^2/m^2 in men with a body mass index < 24.9 kg/m^2; < 53 cm2/m^2 in men with a body mass index ≥ 25 kg/m^2; and < 41 cm^2/m^2 in women). N = 44 due to missing data (included); N = 11 due to missing data (excluded).

[5](Probable)-sarcopenia diagnosed according to revised European Working Group on Sarcopenia in Older People 2019 definition (EWGSOP2): probable sarcopenia (handgrip strength < 27 kg (male) and < 16 kg (female)); sarcopenia (low handgrip strength plus low muscle mass); severe sarcopenia (low handgrip strength plus low muscle mass plus Short Physical Performance Battery (SPPB) score ≤ 8). N = 44 due to missing data; N = 11 due to missing data (excluded).
