# Supplementary material for: Diet Quality Is Not Associated with Malnutrition, Low Muscle Mass and Sarcopenia During Lung Cancer Treatment: A Cross-Sectional Study
Source: Nutrients. 2026 Feb 26;18(5):764. doi: 10.3390/nu18050764 (PMC12986464; doi:10.3390/nu18050764)
Supplement: Supplementary file 1 [file nutrients-18-00764-s001.zip › Table S6.pdf]

Supplementary Table S6. Dietary Guideline Index 2013 percentage of non-consumers, component scores and percentage of participants meeting dietary guidelines, overall and by sex: men (n = 27) and women (n = 20)

DGI-2013 components[1]	Non-consumers (%)[2]	DGI-2013 component score[3]			Meeting guideline (%)[4]		
		Total	Men	Women	Total	Men	Women
1. Enjoy a wide variety of nutritious foods		3.90 ± 1.30	3.78 ± 1.25	4.08 ± 1.40	0.0	0.0	0.0
2. Enjoy plenty of vegetables	4.3	5.52 ± 2.94	5.53 ± 3.49	5.52 ± 2.07	14.9	18.5	10.0
3. Enjoy fruit	19.2	4.52 ± 3.13	4.81 ± 3.52	4.11 ± 2.56	4.3	7.4	0.0
4. Enjoy grain (cereal) foods, mostly wholegrain or high cereal fibre varieties							
4a. Total cereals intake	29.8	1.57 ± 1.35	1.32 ± 1.2.9	1.90 ± 1.38	2.1	3.7	5.0
4b. Mostly wholegrain	57.5	1.75 ± 2.42	1.96 ± 2.50	1.47 ± 2.35	35.0	39.1	29.1
5. Enjoy lean meats, poultry, fish, and alternatives							
5a. Total meats intake	57.4	1.69 ± 2.10	2.13 ± 2.07	1.10 ± 2.05	17.0	14.8	20.0
5b. Mostly lean[3]	17.0	3.23 ± 1.95	3.50 ± 1.84	2.41 ± 2.27	40.0	46.7	20.0
6. Enjoy milk, yoghurt, cheese and alternatives, mostly reduced fat	23.4	2.13 ± 1.91	1.72 ± 1.75	2.69 ± 2.02	0.0	0.0	0.0
7. Drink plenty of water							
7a. Total beverages intake	6.4	1.02 ± 0.76	1.02 ± 0.85	1.01 ± 0.64	0.0	0.0	0.0
7b. Mostly water	25.5	3.79 ± 2.14	3.03 ± 2.45	5.00 ± 0.00	75.0	59.3	100.0

8. Limit intake of foods containing saturated fat, added salt, added sugars and alcohol	2.1	5.96 ± 4.96	6.30 ± 4.92	5.50 ± 5.10	59.6	63.0	55.0
9. Limit intake of food high in saturated fat							
9b. Mostly low-fat milk	85.1	0.47 ± 1.48	0.00 ± 0.00	1.00 ± 2.07	9.4	0.0	20.0
10. Small allowance of unsaturated fats, oils and spreads	53.2	7.66 ± 4.28	8.15 ± 3.96	7.00 ± 4.70	76.6	81.5	70.0
11. Limit intake of foods and drinks containing added sugars	12.8	4.47 ± 5.03	3.70 ± 4.92	5.50 ± 5.10	44.7	37.0	55.0
12. Limit intake of alcohol	72.3	9.36 ± 2.47	9.26 ± 2.67	9.50 ± 2.24	93.6	92.6	95.0
**Total DGI**		53.00 ± 13.00	52.96 ± 14.04	53.13 ± 11.72	0.0	0.0	0.0

Abbreviations: *DGI-2013, Dietary Guideline Index 2013*

[1]*DGI components were scores 0-10, sub-components were scored 0-5, total DGI-2013 scores range was 0-115.*

[2]*Percentage on non-consumers is the proportion who consumed 0 serves of the DGI component.*

[3]*Values represent mean ± SD.*

[4]*Those with a maximum DGI component score were considered meeting the guideline.*
