# Supplementary material for: Diet Quality Is Not Associated with Malnutrition, Low Muscle Mass and Sarcopenia During Lung Cancer Treatment: A Cross-Sectional Study
Source: Nutrients. 2026 Feb 26;18(5):764. doi: 10.3390/nu18050764 (PMC12986464; doi:10.3390/nu18050764)
Supplement: Supplementary file 1 [file nutrients-18-00764-s001.zip › Table S7.pdf]

Supplementary Table S7. Mediterranean Diet Score percentage of non-consumers, component scores and percentage of participants meeting dietary guidelines, overall and by sex: men (n = 27) and women (n = 20)

Mediterranean Diet Score components[1]	Non-consumers (%)[2]	Mediterranean Diet Score component score[3]			Meeting recommendations (%)[4]		
		Total	Men	Women	Total	Men	Women
1. Vegetables	4.3	0.51 ± 0.51	0.52 ± 0.51	0.50 ± 0.51	51.1	51.9	50.0
2. Legumes[5]	89.4	0.06 ± 0.25	0.07 ± 0.27	0.05 ± 0.22	6.4	7.4	5.0
3. Fruits and nuts	17.0	0.51 ± 0.51	0.52 ± 0.51	0.50 ± 0.51	51.1	51.9	50.0
4. Cereals and grains	0.0	0.51 ± 0.51	0.52 ± 0.51	0.50 ± 0.51	51.1	51.9	50.0
5. Fish and seafood[5]	63.8	0.26 ± 0.44	0.19 ± 0.40	0.35 ± 0.49	25.5	18.5	35.0
6. Mono-unsaturated fat: saturated fat ratio		0.51 ± 0.51	0.52 ± 0.51	0.50 ± 0.51	51.1	51.9	50.0
7. Dairy products	10.6	0.49 ± 0.51	0.48 ± 0.51	0.50 ± 0.51	48.9	48.2	50.0
8. Meat and meat products	2.1	0.51 ± 0.51	0.52 ± 0.51	0.50 ± 0.51	51.1	51.9	50.0
9. Alcohol	72.3	0.21 ± 0.41	0.15 ± 0.36	0.30 ± 0.47	21.3	14.8	30.0
**Total Mediterranean Diet Score**		3.6 ± 1.5	3.5 ± 1.5	3.7 ± 1.7	0.0	0.0	0.0

[1]*Mediterranean Diet Score components were scored 0-1, where 1 was scored if consumption was above the median (by sex) intake by sex for positive components (vegetables, legumes, fruits and nuts, cereals and grains, fish and seafood and monounsaturated fat ratio) and below the median (by sex) for negative components (dairy products, meat and meat products). For alcohol, 1 was scored if intake was above zero but no more than two serves per day (600kJ per serve). Total Mediterranean Diet Scores ranged from 0-9.*
[2]*Percentage on non-consumers is the proportion who consumed 0 serves of the Mediterranean Diet Score component.*
[3]*Values represent mean ± SD.*

[4]Those who consumed above the sex-specific median (score of 1 for each component) were considered meeting the guideline.
[5]Due to low consumption rates of legumes and fish and seafood components, median cut-offs were derived from consumers.
