# Supplementary figures and images for: Diet Quality Is Not Associated with Malnutrition, Low Muscle Mass and Sarcopenia During Lung Cancer Treatment: A Cross-Sectional Study
Source: Nutrients. 2026 Feb 26;18(5):764. doi: 10.3390/nu18050764 (PMC12986464; doi:10.3390/nu18050764)

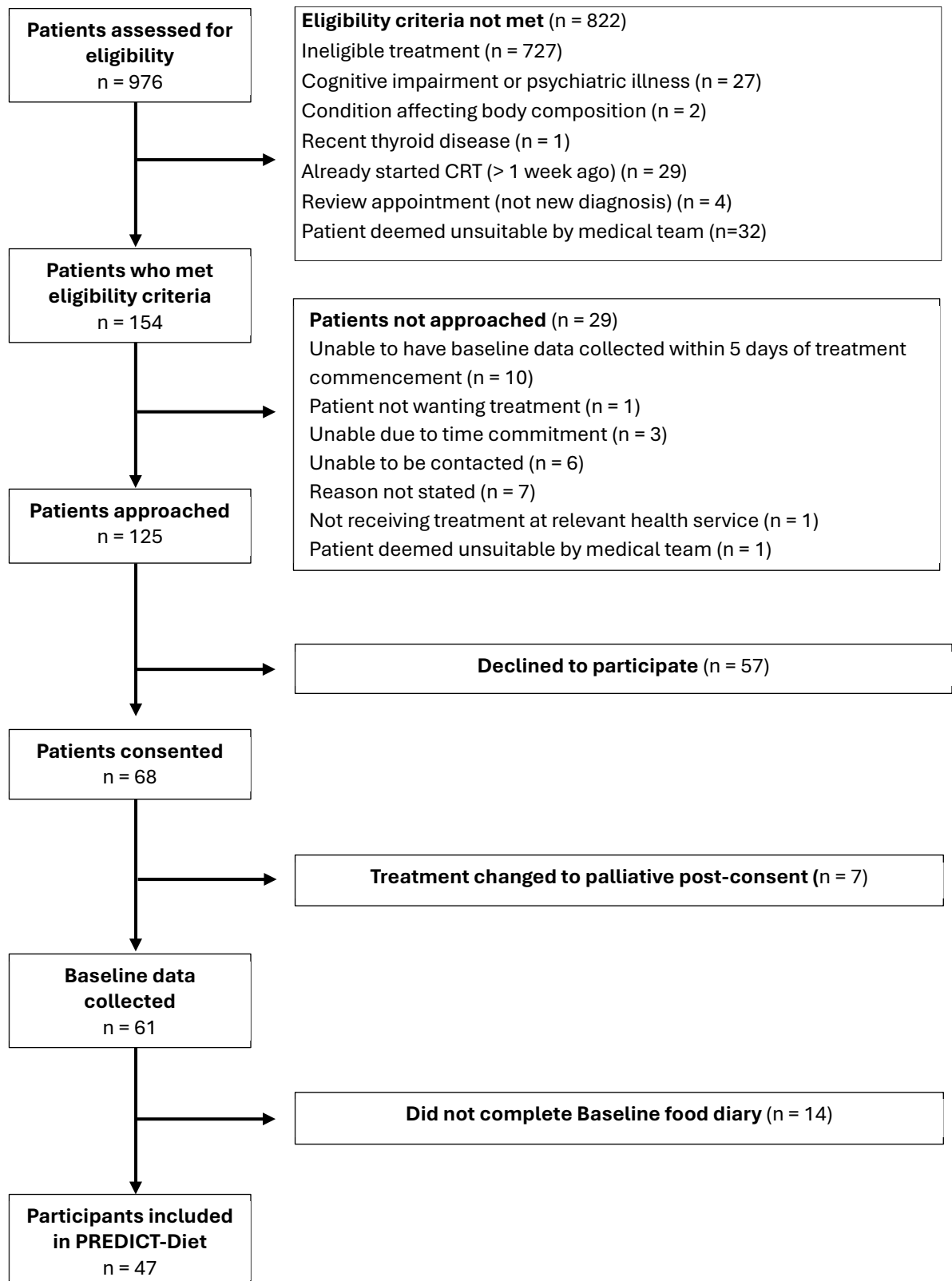

**Supplementary Figure S2.** Flow diagram of eligible participants from the PREDICT Study

Supplement: Supplementary file 1 [file nutrients-18-00764-s001.zip › Figure S2.pdf]
